# miR-133: A Suppressor of Cardiac Remodeling?

**DOI:** 10.3389/fphar.2018.00903

**Published:** 2018-08-17

**Authors:** Ning Li, Heng Zhou, Qizhu Tang

**Affiliations:** ^1^Department of Cardiology, Renmin Hospital of Wuhan University, Wuhan, China; ^2^Cardiovascular Research Institute, Wuhan University, Wuhan, China; ^3^Hubei Key Laboratory of Cardiology, Wuhan, China

**Keywords:** miR-133, fibrosis, hypertrophy, electrical remodeling, reprogram

## Abstract

Cardiac remodeling, which is characterized by mechanical and electrical remodeling, is a significant pathophysiological process involved in almost all forms of heart diseases. MicroRNAs (miRNAs) are a group of non-coding RNAs of 20–25 nucleotides in length that primarily regulate gene expression by promoting mRNA degradation or post-transcriptional repression in a sequence-specific manner. Three miR-133 genes have been identified in the human genome, miR-133a-1, miR-133a-2, and miR-133b, which are located on chromosomes 18, 20, and 6, respectively. These miRNAs are mainly expressed in muscle tissues and appear to repress the expression of non-muscle genes. Based on accumulating evidence, miR-133 participates in the proliferation, differentiation, survival, hypertrophic growth, and electrical conduction of cardiac cells, which are essential for cardiac fibrosis, cardiac hypertrophy, and arrhythmia. Nevertheless, the roles of miR-133 in cardiac remodeling are ambiguous, and the mechanisms are also sophisticated, involving many target genes and signaling pathways, such as RhoA, MAPK, TGFβ/Smad, and PI3K/Akt. Therefore, in this review, we summarize the critical roles of miR-133 and its potential mechanisms in cardiac remodeling.

## Introduction

Heart failure (HF), a rapidly growing public health issue, had an estimated prevalence of over 37.7 million individuals worldwide in 2016, contributing to a grave disease burden (Ziaeian and Fonarow, 2016). As a ubiquitous clinical manifestation of various end-stage heart diseases, HF is distinguished by interstitial fibrosis, chamber remodeling, reduced ventricular compliance, and abnormal electrical conduction (Chamberlain et al., 2015; Lee et al., 2015). Cardiac remodeling is one of the decisive pathophysiological processes, exhibiting multiple roles in HF (Saba et al., 2008). Cardiac fibrosis, hypertrophy, and electrical remodeling are the most important physiological or pathological changes observed in cardiac remodeling and directly affect the cardiac function and even cause death (Tao et al., 2016). Therefore, investigations of the associations between cardiac remodeling and risk factors, as well as the molecular mechanisms are important and will contribute to the development of novel and effective approaches to protect against HF.

MicroRNAs (miRNAs) are a class of small non-coding RNAs of approximately 20-25 nucleotides in length that are typically excised from putative miRNA precursor (pre-miRNA) with 60–110 nucleotides and play vital biological roles by directly binding to the 3′ UTR of target messenger RNAs (mRNAs) (Pan et al., 2017). By pairing with target mRNAs encoding proteins, miRNAs could regulate the expression of these mRNAs at the post-transcriptional levels (Calin and Croce, 2006), in addition to affecting the stability and/or the translation of mRNA (Xiao, 2011). Many miRNAs participate in various crucial biological processes in the human body, including proliferation, differentiation, development, and cell apoptosis (Vidigal and Ventura, 2015). Based on accumulating evidence, miRNAs function as important regulators of cardiovascular disorders, such as hypertension (Courboulin et al., 2011), stroke (Jeon et al., 2013), atrial fibrillation (AF) (Goren et al., 2014), left ventricular hypertrophy and HF (Wronska et al., 2015). Using cardiac fibrosis as an example, miR-22 may inhibit angiotensin II (Ang II)-induced cardiac fibrosis and the transformation of cardiac fibroblasts to myofibroblasts by suppressing the expression of transforming growth factor β(TGF-β) receptor I (Tβ RI) in the heart (Hong et al., 2016). Moreover, miR-101a also inhibits the expression of Tβ RI and its downstream molecules to suppress differentiation, migration and collagen synthesis of cardiac fibroblasts (Zhao et al., 2015). However, miRNAs also act as accomplices in cardiovascular disorders associated with cardiac fibrosis. The miR-34 cluster, particularly miR-34a, is up-regulated in the heart following myocardial infarction (MI). By directly targeting Smad4, miR-34a exacerbates the progression of cardiac fibrosis, suggesting that inhibition of miR-34a may be a promising strategy to treat cardiac fibrosis (Huang et al., 2014). Additionally, miR-21 has been also shown to promote the transduction of extracellular signal-regulated kinase/extracellular signal-regulated kinases—mitogen-activated protein kinase (ERK-MAPK) signals, thus aggravating cardiac fibrosis and deteriorating cardiac function (Thum et al., 2008). Hence, different miRNAs may exert diverse effects on cardiac remodeling, but the underlying mechanisms remain unclear.

In the present review, we synthesize the roles and mechanisms by which miR-133 protects against cardiac remodeling to provide logical evidence for further investigations and clinical applications in the future.

## Cardiac remodeling

Cardiac remodeling is generally accepted as constant adjustments of the myocardium to the structure, metabolism and electrical conduction under the influence of various endogenous and exogenous factors, which eventually result in alterations in the ventricle structure and biological effects (Cohn et al., 2000; Zhou et al., 2013). Generally, cardiac remodeling is classified as mechanical remodeling and electrical remodeling, according to the mechanisms by which this process contributes to cardiac dysfunction (Jeyaraj et al., 2007; Lellouche et al., 2011). Depending on the type of cardiac cells exhibiting pathological changes, mechanical remodeling is further classified as cardiac fibrosis and cardiac hypertrophy (Gerdes and Capasso, 1995; Kamireddy et al., 2009).

### Mechanical remodeling

In cardiac fibrosis, the excessive deposition of extracellular matrix (ECM) proteins secreted by cardiac fibroblasts reduces tissue compliance and accelerates the progression of HF (Travers et al., 2016). Various quantitative and qualitative changes in the interstitial collagen network, which usually occur to enable the cardiac tissue to adapt to ischaemic insults, drugs, systemic diseases, or any other harmful stimulus that affects the heart itself and the circulatory system, can trigger cardiac fibrosis (Travers et al., 2016). The harmful stimulus induces cardiac fibroblasts to transiently express α-smooth muscle actin (α-SMA), an actin isoform that is a marker for myfibroblast and mainly responsible for the homeostasis of the ECM (Hinz et al., 2001). The α-SMA protein in cardiac fibroblasts is connected to the ECM through focal adhesions, and is assembled into stress fibers and remodels the surrounding ECM in response to certain types of stress, eventually causing HF (Leask, 2015; Tian et al., 2017). Although several studies have explored the origin of cardiac fibroblasts, accurate answers are still unavailable. Nevertheless, cardiac fibroblasts are generated from multiple sources, such as the proliferation of existing fibroblasts, the recruitment of pericyte-like progenitor cells, differentiation of resident mesenchymal cells mediated by growth factors or even the epithelial-mesenchymal transition (EMT) (Hinz et al., 2012). In recent years, the cellular microenvironment was also disclosed to play a vital role in enhancing the pathological responses of cardiac fibroblasts to growth factors/cytokines and hormones during cardiac fibrosis (Gao et al., 2016).

Cardiac hypertrophy is broadly defined as an increase in the myocardial mass to continuously pump blood to provide the organism with nutrients and oxygen (Sayed et al., 2007). It is one of the critical ways in which cardiomyocytes respond to physiological or pathological haemodynamic overload and neurohormonal stimuli, such as chronic exercise training, regular physical activity and gestation (physiological) or valve disease, hypertension, HF, and MI (pathological) (Lyon et al., 2015; Shimizu and Minamino, 2016). Both physiological and pathological cardiac hypertrophy may lead to an increase in cardiomyocyte size to improve their functional output and maintain cardiac function, without altering the number of cardiomyocytes (Tham et al., 2015). Notably, physiological hypertrophy is reversible and is characterized by normal cardiac morphology (no apoptosis and fibrosis) and normal or enhanced cardiac function (Kamo et al., 2015). Conversely, pathological hypertrophy, which exhibits the hallmarks of increased protein synthesis and alterations in the organization of sarcomere structure in cardiomyocytes, is generally followed by deleterious events involving cardiac dysfunction, cardiac fibrosis, oxidative stress, endoplasmic reticulum stress and fetal gene upregulation, including genes that encode brain natriuretic peptide (BNP), atrial natriuretic peptide (ANP), and β-myosin heavy chain (β-MHC) (Bernardo et al., 2010; Huang et al., 2013; Gao and Wang, 2016). Initially, cardiac hypertrophy is an adaptive response to maintain cardiac output. However, abnormal growth may gradually spread to one chamber of the heart, one side of the heart, and even the entire structure of heart uniformly because of pressure or volume overload and other hormonal or cytokine stimuli, which may eventually lead to HF or sudden death (Morgan and Baker, 1991; Heineke and Molkentin, 2006).

**Figure 1:**
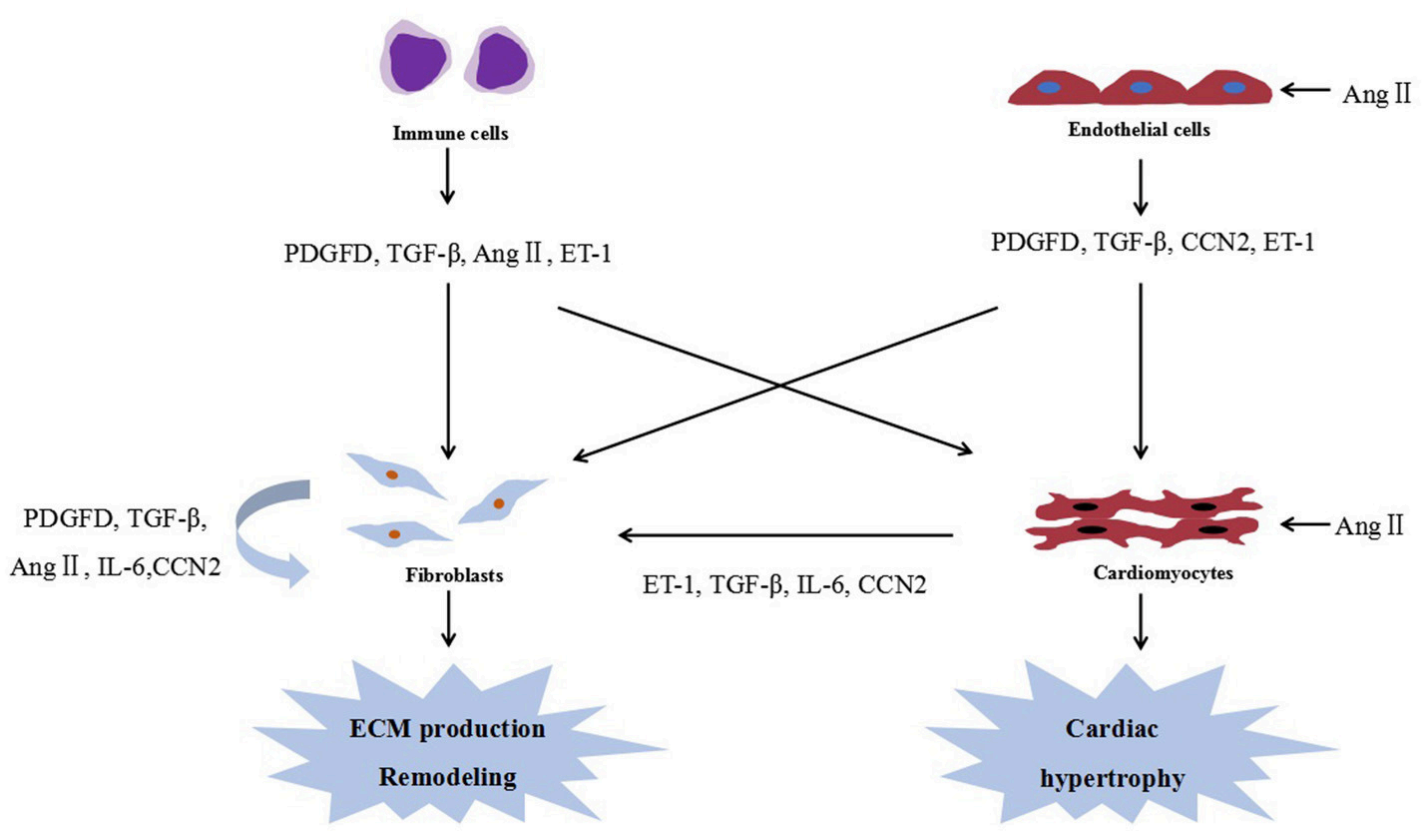
Cellular interactions in the heart. Ang II, angiotensin II; PDGFD, platelet-derived growth factor D; ECM, extracellular matrix; ET-1, endothelin-1; IL-6, interleukin 6; and TGF-β, transforming growth factor-β.

To date, several studies examining the interplay between cardiac fibrosis and cardiac hypertrophy have focused on cytokines secreted by cardiac fibroblasts and cardiomyocytes. Figure 1 shows the cellular interactions in the heart. In fact, many of these cytokines could be modulated by miRNAs, and are expected to be their potential targets.

### Electrical remodeling

Electrical remodeling refers to alterations in the electrophysiological milieu of the myocardium in patients with various cardiac disorders, such as HF, ventricular hypertrophy, myocardial ischaemia, and infarction, which are commonly associated with cardiac arrhythmias (Mueller et al., 2011; Aguilar et al., 2014). Electrical remodeling is distributed into four classes, depending on the level at which remodeling occurs, including ionic channel remodeling, cardiomyocyte electrical remodeling, myocardial electrical remodeling, and cardiac conduction system electrical remodeling (Mitchell et al., 1993; Lee et al., 2017). Generally, the initial rapid phase and the terminal phase of ventricular repolarization in cardiomyocytes are triggered by a transient outward K^+^ current (I_to_) and inward rectifier K^+^ current (I_K1_), respectively (Chen et al., 2017). In patients with HF and experimental HF models, the current densities of I_K1_ and I_to_ are significantly reduced, indicating that the reduction of I_K1_ and I_to_ plays a vital role in abnormal ventricular membrane repolarization (Zicha et al., 2004). These alterations might cause adverse ionic remodeling and result in an excess prolongation of the action potential duration, which eventually contribute to malignant arrhythmias (Huo et al., 2014). In essence, either functional impairments or alterations in the expression of channel proteins responsible for these ion currents may result in pathological decreases in these current densities in the heart (Marczenke et al., 2017). Hence, strategies that inhibit the downregulation of these ion channels are very important for preventing HF.

Taken together, cardiac remodeling is a sophisticated pathophysiological process in HF and involves many types of cells, molecules, and signaling pathways, which represent numerous potential miRNA targets, thus miRNAs may modulate any of the links in the cardiac remodeling process to ameliorate or aggravate disease conditions.

## The biogenesis, structure and targets of miR-133

**Figure 2:**
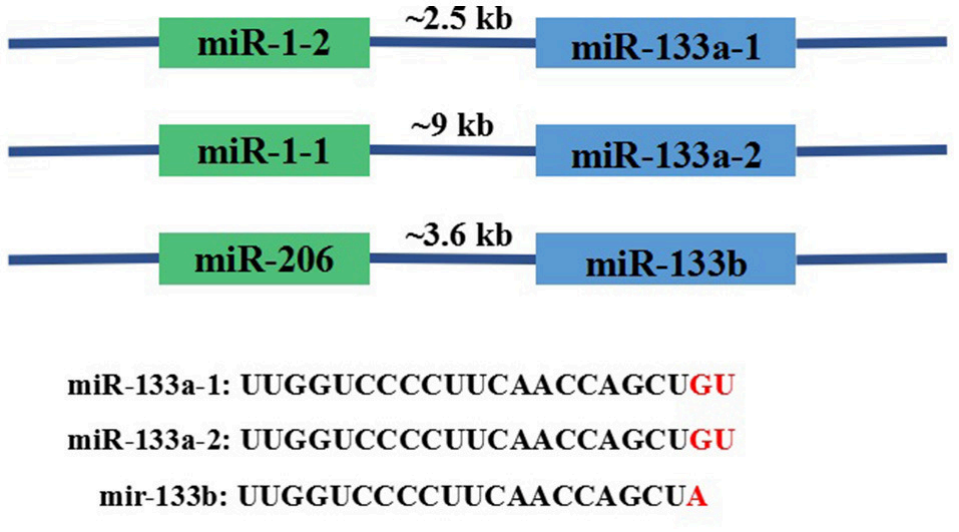
Basic information about the genomic organization of the miR133 family in mice. As shown in the figure, miR-133a-1, miR-133a-2, and miR-133b are transcribed as bicistronic transcripts with miR-1-2, miR-1-1, or miR-206. Genomic distances between the miRNA coding regions are 2.5, 9, and 3.6 kb, respectively.

MiR-133 was first experimentally characterized in mice and its homologs were identified in several other species, including invertebrates such as the fruit fly *Drosophila melanogaster* (Chen et al., 2006). In the human genome, miR-133 genes include miR-133a-1, miR-133a-2, and miR-133b located on chromosomes 18, 20, and 6, respectively. Importantly, miR-133a-1 and miR-133a-2 have identical nucleotide sequences, whereas miR-133b differs in the last 2 nucleotides (GU → A) at the 3′ terminus. The three miRNAs are transcribed as a bicistronic transcript with miR-1-2, miR-1-1, or miR-206, respectively, as shown in (Figure 2; Ohanian et al., 2013). Ten variants of miR-1 and miR-133 were identified in 120 individuals with familial atrial fibrillation (AF). The miR-133a-2 haplotype comprises six nucleotide changes, namely, −19G > A; −102G > A; −82G > A; 79T > C; +69G > A; and +47T > C. The miR133B haplotype only contains one variation, −25delA. The miR-1 haplotype comprises three nucleotide changes, −43C > T, +15A > G, and +74T > C (Ohanian et al., 2013). The sites in which miR-133a and miR-133b are expressed also differ; the bicistronic miR-1/miR-133a cluster is expressed in both skeletal and cardiac muscle, while the miR-206/miR-133b cluster is mainly expressed in skeletal tissue (Ivey et al., 2008; Liu et al., 2008). Many crucial myocyte differentiation factors, such as myocyte enhancer factor-2 (Mef2), serum response factor (SRF), myogenic differentiation-1 (MyoD), and myocardin, regulate the transcription of miR-1 and miR-133a (Zhao et al., 2005).

The miR-133 genes appear to be dysregulated in many human diseases, including gastric cancer (Yang et al., 2017), non-small cell lung cancer (Wang Y. et al., 2016), pituitary tumors (Wang D. S. et al., 2016), and various cardiovascular diseases (Liu et al., 2017). Insulin has been shown to regulate miR-133 expression. The insulin treatment triggers the translocation of active SREBP-1c from the endoplasmic reticulum to the nucleus, which concomitantly induces SREPB-1c expression via the PI3K signaling pathway. Consequently, the elevated level of active SREPB-1c suppresses the expression of MEF2C, a protein that binds to the enhancer region of miR-133. Due to the lower MEF2C expression, the transcription of miR-133a is reduced, eventually leading to decreased levels of its mature forms in heart and muscle (Granjon et al., 2009). Additionally, the gene polymorphism of miR-133 is also implicated with the metabolism of drugs. Pérez-Andreu et al. (2012) identified the potential binding sites for miR-133 in human hepatocytes and found that it was involved in regulating VKORC1 expression, a target of warfarin, in response to anti-coagulant treatment. Regarding the warfarin dose, patients carrying the rs45547937 A allele of the miR-133a-2 gene required a significantly higher warfarin dose to achieve the therapeutic benefit than patients carrying the rs9923231 wild-type allele (Ciccacci et al., 2015).

As reported, miR-133 is predominantly expressed in cardiomyocytes and cardiac fibroblasts in the heart, and the overexpression, targeted deletion, or antisense-specific knockdown of miR-133 genes have revealed many of its functions and targets in cardiac remodeling (Townley-Tilson et al., 2010). For instance, in the study by Sang et al. a miR-133a mimic and miR-133a overexpression significantly decreased fibrosis in the heart by inhibiting serine/threonine kinase Akt in rats with chronic HF (Sang et al., 2015). During cardiac reprogram, miR-133 overexpression also suppresses the expression of a large number of genes in fibroblasts and concomitantly activates cardiac gene programme (Muraoka et al., 2014). Additionally, the downregulation of miR-133a expression results in the ectopic expression of smooth muscle genes in myocardium and aberrant cardiomyocyte proliferation by activating SRF, a positive cardiomyocyte-specific growth and differentiation factor that binds to the CArG boxes in the regulatory region (Liu et al., 2008). More recently, miR-133 was proven to modulate multiple components of the β-adrenergic receptor (β-AR) signal transduction cascade to protect against cardiac fibrosis (Wang D. et al., 2017). Table 1 summarizes the abnormal expression of miR-133 genes and their targets observed in cardiac remodeling.

**Table 1:** miR-133 and verified target genes involved in cardiac remodeling.

**Tissue/cells**	**miR-133 level**	**Target gene**	**Model**	**References**
Cardiomyocytes	Down	RhoA, Cdc42, Nelf-A/WHSC2	Mice and humans with cardiac hypertrophy	Carè et al., 2007
Cardiomyocytes	Down	Cyclin D2	miR-133a double-mutant mouse	Liu et al., 2008
Cardiomyocytes	Down	Caspase-3	Infarcted rats	Xu et al., 2014
Vascular smooth muscle cells	Down	Sp-1	Rats with balloon injury of the right carotid artery	Torella et al., 2011
Cardiomyocytes and fibroblasts	Down	Ctgf	Rats with hypertension-induced heart failure	Duisters et al., 2009; Angelini et al., 2015;Li et al., 2016
Cardiomyocytes	Down	IGF-1	Mouse model of cardiac hypertrophy	Hua et al., 2012
HEK293 cells	Down	IP_3_RII	Mouse model of cardiac hypertrophy	Drawnel et al., 2012
HEK293 cells	Down	Calcineurin	Rat model of pressure-overload cardiac hypertrophy	Dong et al., 2010
Atrial fibroblasts and atrial tissues	Down	TGF-β	Canine model of atrial fibrosis	Shan et al., 2009
Embryonic fibroblasts	Up	Snai1	Mouse	Muraoka et al., 2014
Cardiac fibroblasts	Down	Collagen 1a1	Rats with cardiac fibrosis induced by angiotensin II	Castoldi et al., 2012
Cardiac fibroblasts	down	FGF1	Mice with diabetic cardiomyopathy	Chen et al., 2014
Mesenchymal stem cells	Transfected	Apaf-1	Infarcted rats	Dakhlallah et al., 2015
Cardiomyocytes	Up	KCNH2	Guinea pig model of As_2_O_3_-induced QT prolongation	Shan et al., 2013
Ventricular myocytes	Down	Kcnip2	Adult mouse hearts subjected to pressure overload	Matkovich et al., 2010
Heart/HEK293 cells	Up	HERG	Rabbit model of diabetic cardiomyopathy	Xiao et al., 2011
Cardiac progenitor cells	Up	Bmf and Bim	Mouse	Li R. et al., 2010
Zebrafish heart/cardiomyocytes	Down	Cx43	Zebrafish model with partial heart resection	Yin et al., 2012
Rat ventricular cardiomyocytes	Down	NFATc4	Rats with pressure overload-induced cardiac hypertrophy	Li Q. et al., 2010
C2C12 myoblasts	Transfected	FGFR1 and PP2AC	C2C12 cell lines	Feng et al., 2013

## The roles of miR-133 in cardiac remodeling

**Figure 3:**
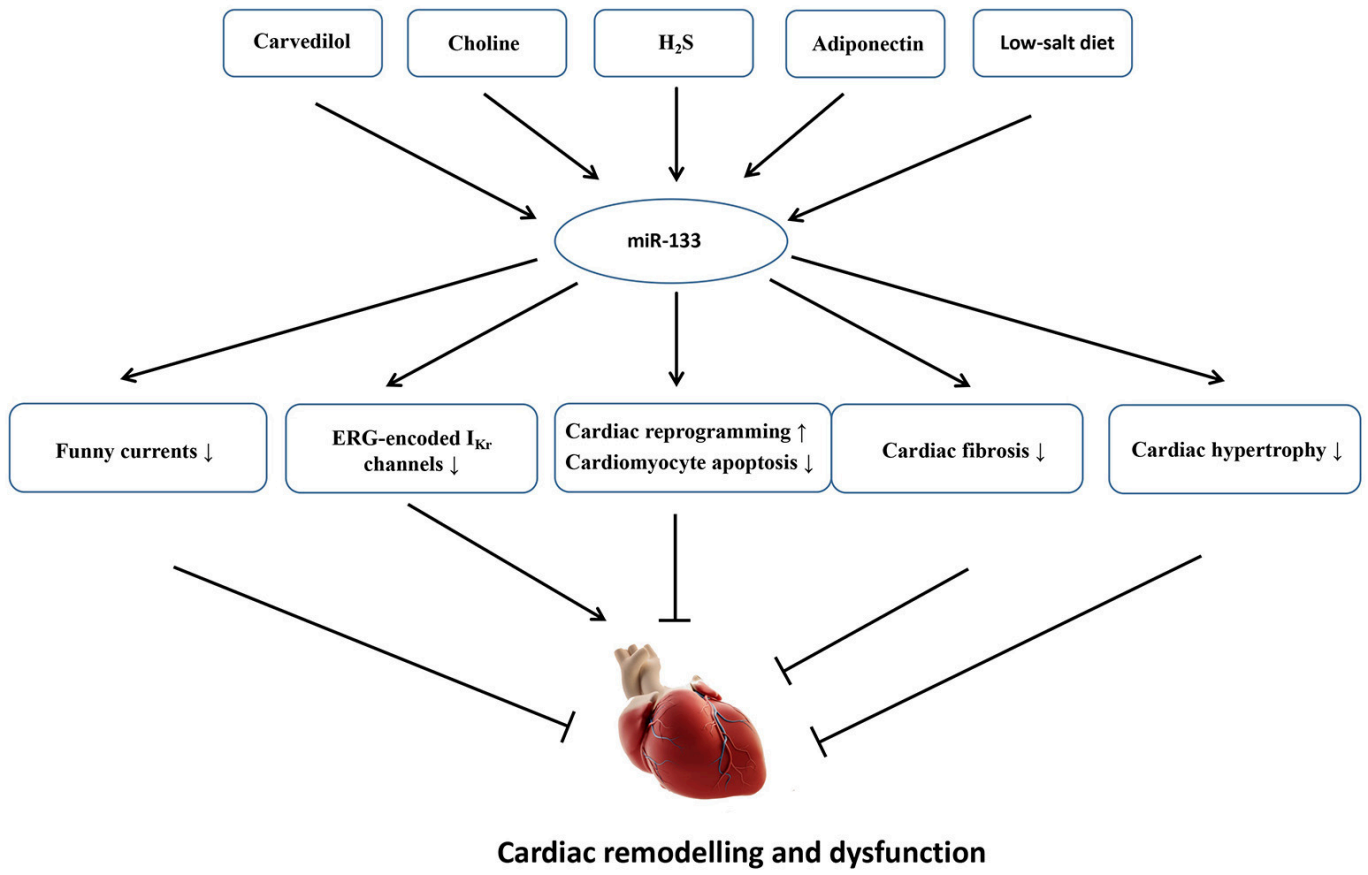
An overview of the roles of miR-133 in cardiac remodeling as well as some endogenous and exogenous molecules that are verified to regulate miR-133 expression. Carvedilol, choline, H_2_S, adiponectin, and low-salt diet upregulate miR-133 expression. The increased miR-133 could inhibit cardiac fibrosis, cardiac hypertrophy, funny currents, and cardiomyocyte apoptosis and contribute to cardiac reprogram, which will mitigate cardiac dysfunction and remodeling. On the other hand, the increased miR-133 also suppresses ERG-encoded IKr channels, eventually aggravating electrical remodeling.

MicroRNAs are atypically expressed in the cardiovascular system under some specific pathological conditions; the expression levels of miRNAs are usually altered when the myocardium is subjected to cardiac stress/insult (Da Costa Martins and De Windt, 2012). Although miR-133 is mainly expressed in the myocardium, and other copies of miR-133 have not been identified within the genome, miR-133 is dispensable for the heart development and cardiac morphogenesis because cardiac-specific or global deletion of miR-133 did not give rise to premature death or cardiac abnormalities in mice, and miR-133 null mice display normal fertility and viability. By contrast, miR-133 is essential for cardiac remodeling in response to different stresses (Matkovich et al., 2010). In a clinical study, the decreased expression of miR-133 was significantly associated with the severity of patients with HF. Patients with a high NT-proBNP concentration (>1,800 pg/ml) showed a 25% decrease in miR-133 expression compared with patients with a low concentration (< 300 pg/ml) (*p* = 0.023) (Danowski et al., 2013). In animal models, miR-133 also exerts multiple functions to regulate the development of cardiac fibrosis, cardiac hypertrophy, electrical activities and cardiac reprogram. Figure 3 shows an overview of the roles of miR-133 in cardiac remodeling as well as some endogenous and exogenous molecules that are verified to regulate miR-133 expression.

### Inhibiting the progression of cardiac fibrosis

Cardiac fibrosis is distinguished by the excess deposition of ECM and the transformation of cardiac fibroblasts to myofibroblats; the deposition and the transformation negatively impact organ architecture and function (Wu et al., 2017b). A network of cytokines could regulate cardiac fibrosis, and abnormal activation of cardiac fibrosis is linked to various cardiac disorders through numerous molecular pathways and proteins. In particular, the transforming growth factor (TGF)-β/Smad pathway, mitogen-activated protein kinase (MAPK) pathway and PI3K/Akt pathway are several canonical signaling pathways participating in cardiac fibrosis.

#### TGF-β/Smad pathway

The TGF-β/Smad pathway has been implicated in many fibrotic disorders, including liver cirrhosis (Kim et al., 2011), glomerulonephritis (Fukasawa et al., 2004), lung fibrosis (Yu et al., 2008), and vascular restenosis (Akiyama-Uchida et al., 2002). The TGF-β/Smad pathway can regulate the endothelial-mesenchymal transition (EndMT), meanwhile it is activated by the sarcomere protein mutation (α-myosin heavy chain, α-MHC R719W) (Dijke and Hill, 2004). Under pathological conditions, the EndMT displays a critical role in the production of collagen and is predominantly regulated by signaling pathways mediated by cytokines, including TGF-β (Yoshimatsu and Watabe, 2011). Connective tissue growth factor (CTGF), a cysteine-rich protein whose expression is induced by TGF-β through a signaling cascade requiring Smad and protein kinase C (PKC) in connective tissue cells, acts as a key regulator of cardiac fibrosis, which could give rise to collagen synthesis (Guo et al., 2015; Huang M. et al., 2016). On the other hand, the expression of mutant sarcomere proteins in myocyte cells also changes gene transcription in non-myocyte cells and contributes to the expression of profibrotic molecules, eventually inducing pathological remodeling in hypertrophic cardiomyopathy (Gong et al., 2011). In pathological remodeling, the activation of the TGF-β/Smad pathway in non-myocyte is a pivotal mechanism underlying the increased fibrosis in patients with hypertrophic cardiomyopathy and a potentially important factor causing diastolic dysfunction and HF (Teekakirikul et al., 2010). Additionally, TGF-β1 directly blocks PPARγ expression in cardiac fibroblasts at the transcriptional level, indicating that the down-regulation of endogenous proliferator-activated receptor γ (PPARγ) expression by TGF-β is a key regulatory mechanism in cardiac fibrosis (Gong et al., 2011). Based on the findings from aforementioned experimental studies, we can conclude that the TGF-β/Smad signaling pathway possesses a vital role in the cardiac remodeling process, particularly in cardiac fibrosis. In a study by Chen and colleagues examining a mouse model of diabetic cardiomyopathy, miR-133a significantly decreased the expression of TGF-β1 and increased the expression of acidic fibroblast growth factor1 (FGF1) that could induce ERK1/2 phosphorylation (Chen et al., 2014). In atrial fibroblasts, however, cigarette smoking/nicotine exposure activated the α7 nicotinic acetylcholine receptor (α7-nAChR) and decreased the expression of miR-133, thus increasing the expression of TGF-β1 and resulting in enhanced collagen production as well as fibrosis generation (Shan et al., 2009). Apart from targeting TGF-β1, in fact, miR-133a could also bind to the 3′ UTR of the CTGF mRNA and directly down-regulates the expression of CTGF, the downstream molecule of TGF-β/Smad pathway. In patients with severe valvular aortic stenosis and rodent models of pressure overload, the level of miR-133 was inversely correlated with CTGF expression (Duisters et al., 2009; Gjymishka et al., 2016). However, a high level of SRF blocked the inhibitory effect of miR-133 on CTGF expression, leading to the counteraction of the protective effect of miR-133. Intriguingly, a low level of serum response factor (SRF) promotes the protective effect of miR-133 on cardiac fibrosis (Angelini et al., 2015). The potential mechanisms warrant thorough investigation in future studies.

#### Histone deacetylases

Histone acetylation/deacetylation is a vital pathway in regulating gene expression, and the balance between these modifications is mediated by histone acetyltransferases (HATs) and histone deacetylases (HDACs), respectively (Backs and Olson, 2006). Essentially, HDACs and HATs AT are the enzymes that are principally responsible for removing and adding the acetyl moiety to lysine residues of histone proteins, respectively (Lane and Chabner, 2016). Currently, the roles of HDACs in cardiac fibrosis initiation, progression as well as the therapeutic effects of HDACs inhibitors have been well investigated (Akiyama-Uchida et al., 2002; Williams et al., 2014). The function of HDACs in regulating pathological cardiac remodeling was initially verified based on the interaction between class IIa HDACs and members of the myocyte enhancer factor-2 (MEF2) transcription factor family, which are critical regulators of cardiac hypertrophy. Class IIa HDACs could inhibit stress-dependent cardiac remodeling through their interaction with the MEF2 transcription factor (Konno and Seidman, 2010). Protein kinase D (PKD), a stress-responsive kinase, phosphorylates class II HDACs and promotes the dissociation of HDACs from MEF2 to induce the transcription of target genes (Fielitz et al., 2008). Hence, the PKD/class II HDACs/MEF2 axis is of critical importance in the progression of cardiac fibrosis. HDACs are present on miR-133a enhancer regions. HDAC inhibitors (Suberoylanilide Hydroxamic Acid) obviously prevent the pressure overload-induced increase in levels of the CTGF protein and collagen deposition through the interaction with deacetylation of transcription factors, co-activators or co-repressors on the miR-133a enhancer and subsequent blockade of its downstream fibrotic genes (Renaud et al., 2015). Homeodomain-only protein (HOP) is a SRF co-factor that inhibits the transcriptional activity of SRF by recruiting a co-repressor complex including HDACs (Kook et al., 2003). Hence, we postulate that both HOP and HDACs regulate miR-133 and may represent an innovative therapeutic approach to reset the epigenome in patients with cardiac fibrosis. Intriguingly, the effects of HDACs on cardiac fibrosis are also regulated by TGF-β1. More specifically, the TGF-β1 treatment increases the binding of Smad2/3, Smad4 and HDAC1, and reduces the binding of acetylated histone 3 to the PPARγ promoter in cardiac fibroblasts (Gong et al., 2011). As mentioned above, miR-133 inhibits TGF-β1 expression in cardiac fibroblasts to protect against cardiac fibrosis. However, researchers have not determined whether the protective effect is mediated by the inhibition of HDACs.

#### PI3K/Akt pathway

Insulin-like growth factor-1 (IGF-1), a crucial growth factor required for the proliferation, differentiation and survival of cardiomyocytes, mediates physiological cardiac hypertrophy through a PI3K-dependent pathway (Schutte et al., 2016). When tyrosine kinase receptors are bound to and activated by IGF-1 or insulins, the SH2 domain will recruit heterodimers of PI3K containing the regulatory subunit of PI3K, which exhibits high affinity for phosphotyrosine residues in the receptors (Aoyagi and Matsui, 2011). The current studies mainly focus on the class I PI3Ks, comprising the class IA (p110α, β, and δ) and class IB (p110γ, encoded by *Pik3cg*) isoforms (Huang X. et al., 2016). The PI3K/Akt cascade is one of the core signaling pathways, which is usually activated upon IGF-1 receptor stimulation (Stitt et al., 2004). This pathway plays an important role in regulating glucose uptake, metabolism, protein synthesis, and proliferation, all of which share the common goal of maintaining cell survival (Wu et al., 2017b). Mice deficient in p110γPI3 display a more accelerated onset of HF in response to dilated or hypertrophic cardiomyopathy. An elevation in p110αPI3K activity prevents the deterioration of cardiac fibrosis and maintains cardiac function in the pressure-overload model, eventually inhibiting pathological growth (Lin et al., 2010). Previously, miR-133 has been shown to target the PI3K/Akt pathway in various human disorders. For instance, in breast cancer, miR-133a regulates the cell cycle and proliferation during tumorigenesis by targeting epidermal growth factor receptor (EGFR) through the downstream molecule Akt (Cui et al., 2013). Similarly, numerous studies have explored the roles of Akt and Akt-related serine-threonine kinases in signaling cascades that regulate multiple activities in the heart and revealed a requirement for these proteins in the pathogenesis of cardiac fibrosis (Lin et al., 2015; Ying et al., 2017). For example, upregulation of miR-133a significantly decreased in the cardiac fibrosis by inhibiting Akt in patients and rats with chronic HF (Sang et al., 2015). However, the study did not investigate miR-133 levels in cardiac fibroblasts and cardiomyocytes, respectively. Hence, further investigations are warranted to determine whether miR-133 expressed in cardiac fibroblasts or cardiomyocytes plays a leading role in inhibiting cardiac fibrosis in HF models.

#### β-adrenergic receptors

β-Adrenergic receptors (β-ARs) belong to the G protein-coupled receptor family and are genetically and pharmacologically subdivided into three subtypes: β_1_, β_2_, and β_3_ (Nakaya et al., 2012)_._ β-ARs and their guanine nucleotide regulatory protein (G protein)-adenylyl cyclase complex play essential roles in regulating cardiac function (Najafi et al., 2016). The p38 mitogen-activated protein kinase (p38 MAPK) and reactive oxygen species (ROS) also have profound effects on cardiac fibrosis (Lu et al., 2014). The p38 MAPK cascade is activated by multiple receptors, including G-protein-coupled receptors, through the activation of protein kinases A (PKA) and PKC, thereby modulating cell proliferation and collagen synthesis (Zhang et al., 2003). In particular, cardiac fibroblast proliferation could be activated in a ROS/p38 MAPK manner, which is implicated with the production of matrix metalloproteinases (MMPs) and the fibrosis of various organs (Yu et al., 2012). The β-adrenergic system plays vital roles in inducing cardiac dysfunction and ECM remodeling, for the reason that β_2_-AR could regulate ROS/p38 MAPK signaling pathway (Lu et al., 2014). Experimental studies have validated that β-AR and its downstream components, including β-arrestin and the catalytic subunit of 3′,5′ cyclic adenosine monophosphate (cAMP)-dependent PKA, are direct targets of miR-133, which indicates that miR-133 could block the synthesis of collagen fibers by suppressing the β-AR pathway (Wang D. et al., 2017). However, researchers have not determined whether miR-133 exerts a regulatory effect on the other two MAPK signaling pathways (ERK and JNK) in cardiac fibrosis, and thus in-depth studies are required.

Based on these findings, an intricate relationship exists between miR-133 and cardiac fibrosis. Elevated miR-133 expression represses cardiac fibrosis. We propose that these findings only disclose a few functions of miR-133 in cardiac mechanical remodeling. In addition, other signaling pathways and targets related to miR-133 should be topics of further investigation.

### Inhibiting the progression of cardiac hypertrophy

Cardiomyocytes act as a critical role in hypertrophy and remodeling. Various types of mechanical stress and neurohumoral stimuli could increase the size and weight of cardiomyocytes, eventually leading to cardiac hypertrophy (Lyon et al., 2015). The signaling pathways involved in cardiac hypertrophy are complicated and sophisticated, including canonical pathways, such as the MAPK signaling cascade (Wu et al., 2017a), calcineurin-dependent signaling pathway (Molkentin et al., 1998), JAK/STAT signaling pathway (Kodama et al., 1997) and PI3K/Akt signaling pathway (Liu et al., 2007), and non-canonical pathways, such as the Wnt signaling pathway (Takano et al., 2002; Dolinsky et al., 2015). According to recent evidence, many miRNAs participate in the development of cardiac hypertrophy. For instance, miR-10a may markedly inhibit cardiac hypertrophy by targeting Tbx5, an important regulator of cardiac development and the cardiac cell cycle (Zhou et al., 2015; Wang D. et al., 2017). Conversely, miR-451 exacerbates high-fat diet-induced cardiac hypertrophy and lipotoxicity in mouse cardiomyocytes by suppressing the serine/threonine kinase 11 (LKB1)/AMPK signaling pathway (Kuwabara et al., 2015). The expression of miR-133 displays an inverse correlation with the severity of cardiac hypertrophy in different animal models, including exercised rats, transgenic (Tg) mice with selective cardiac overexpression of a constitutively active mutant of the Akt kinase, mice treated with isoproterenol and transverse aortic arch-constricted (TAC) mice (Carè et al., 2007).

#### MAPK

MAPK cascades are triple kinase pathways that include a MAPK kinase (MKK), a MAPK kinase kinase (MKKK), and a terminal MAPK, which are responsible for a diverse repertoire of biological activities, including proliferation, differentiation, metabolism, motility, survival, and apoptosis (Seger and Krebs, 1995). MAPK subfamilies have three family members, namely, c-Jun NH2-terminal kinases (JNK −1, −2, and −3), ERK1/2, p38 kinase (α, β, γ, δ), and big MAPK (BMK or ERK5). The p38 and JNK pathways are mainly activated in response to various stresses, including infections, oxidant stress, cytokines, and osmotic shock, whereas the prototypic ERK1/2 pathway is usually activated in response to growth factor stimulation (Chang and Karin, 2001). In the heart, MAPK signaling critically regulates adaptive and maladaptive cardiac hypertrophy in response to an increased workload or pathological insults. ERK1/2 activation is involved in a beneficial form of cardiac hypertrophy that is potentially advantageous to patients with a dilated or failing heart, while p38 and JNK activation may be implicated in cardiomyopathy (Bueno et al., 2000). Cdc42, a member of the Rho subfamily (Cdc42, RhoA, and Rac1) of small GTP-binding proteins, has well-known roles in actin dynamics and is an upstream activator of MAPK (Han et al., 2017). In fibroblasts, the cell surface receptor Cdo interacts with Bnip-2, thereby activating Cdc42. Then, the activated Cdc42 regulates p38 MAPK activity and myogenic differentiation to link cadherin-based adhesion to the p38α/β MAPK pathway, which enhances the muscle-specific transcriptional programme (Kang et al., 2008). Meanwhile, Cdc42 also acts as a signal transduction kinase associated with MAPK in hypertrophy. During hypertrophy, Cdc42 participates in the rearrangements of cytoskeletal and myofibrillar (Nagai et al., 2003; Liu and Molkentin, 2016). In both neonatal and adult cardiomyocytes, miR-133 overexpression or suppression could result in an elevation or a reduction in Cdc42 levels, respectively. The suppression of hypertrophic growth observed in cells cotransfected with miR-133 and a luciferase reporter gene linked to the wild-type 3′UTR of Cdc42 is mediated by Cdc42 silencing and the subsequently blockade of the MAPK signaling cascade (Carè et al., 2007). Intriguingly, the role of miR-133 in regulating Cdc42 expression in tumors is complex and controversial. For instance, miR-133 is an important negative regulator of Cdc42/P21-activated kinases (PAK pathway) in gastric cancer by silencing Cdc42 expression, which is closely correlated with the tumor size, invasion depth and adjacent organ metastasis (Cheng et al., 2014). In contrast, miR-133 may possess an oncogenic effect and stimulate the progression of cervical carcinoma by downregulating Cdc42 and RhoA (Qin et al., 2012). Here, we hypothesize that miR-133 may also have dual functions in cardiac remodeling via the Cdc42-dependent MAPK pathway. Additionally, study showed that in cardiac hypertrophy, miR-133a might also be downregulated by Ang II through the AMPK and ERK1/2 pathways (Li et al., 2016). However, adiponectin, a cytokine that is predominantly secreted by adipose tissues, reverses hypertrophic growth partially through its positive effect on miR-133a (Li et al., 2016). Furthermore, the suppressive effect of miR-133 on the ERK1/2 signaling pathway in mouse myoblast cells involves an exquisite loop mechanism to regulate myogenesis. In detail, miR-133 represses myoblast proliferation and promotes cell differentiation by inhibiting ERK1/2 activation via targeting PP2AC and FGFR1, while the activated ERK1/2 pathway blocks the expression of miR-133 (Feng et al., 2013). Thus, miR-133 is a promising candidate to treat cardiac hypertrophy because of its modulatory effect on ERK1/2, which are vital proteins involved in remodeling.

#### Calcineurin/NFAT

**Figure 4:**
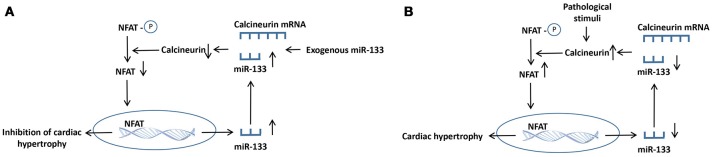
The proposed mechanisms for the reciprocal repression between calcineurin and miR-133 in hypertrophic heart. **(A)** Either exogenous miR-133 supplement or endogenous miR-133 increase gives rise to the transcriptional repression of calcineurin and suppression of NFAT, causing the increased miR-133 level. Eventually, the increased miR-133 further enhanced miR-133 expression. **(B)** The calcineurin/ NFAT signaling is activated by pathological stimuli, giving rise to the downregulation of miR-133 expression. The decreased miR-133 induces the enhancement of calcineurin transcription. Consequently, the activated calcineurin triggers the further increase of calcineurin/ NFAT pathway. ↑, increase; ↓, decrease.

Calcineurin is a member of a class of Ca^2+^-activated serine/threonine phosphatases that is mainly located in the cytoplasm and is modulated by calcium ions in cardiomyocytes (Taigen et al., 2000). Calcineurin was originally identified as an essential component of T lymphocyte signal transduction (Olson and Williams, 2000). In the guinea pig ventricular myocardium, calcineurin reduces the number of gap junctions and connexin levels, allowing action potentials to be propagated among cells by regulating connexin (Cx43) phosphorylation at S365 and S368, eventually decreasing conductivity (Jabr et al., 2016). In the adult heart, calcineurin activity is increased, which precedes the development of pathological cardiac remodeling in response to hypertrophic or failing hearts in humans and pathological insults in mice. Upon activation by calcium ions, calcineurin dephosphorylates the transcription factor nuclear factor of activated T cell (NFAT), leading to nuclear translocation and subsequent hypertrophy (Sun et al., 2016; Yin et al., 2016). Calcineurin is also implicated in the endoplasmic reticulum stress response in cardiomyocyte hypertrophy and apoptosis associated with mitochondrial signaling pathway (Zaja et al., 2014). Several molecules, such as miR-133, LIM, and cysteine-rich domains 1 (Lmcd1), carabin, and PPARγ co-activator 1 α, have been reported to regulate the calcineurin/NFAT signaling pathway during the development of adverse cardiac remodeling (Wu et al., 2017b). In HEK293 cells, the transfection of miR-133 remarkably downregulated calcineurin expression. Meanwhile, this change was reversed by cotransfection with miR-133 inhibitory oligoribonucleotides (Dong et al., 2010). NFATc4, which is one of five NFAT family members, is also one of the direct targets of miR-133a, and its expression is negatively regulated in miR-133a-mediated repression of cardiomyocyte hypertrophy *in vivo* (Li Q. et al., 2010). Surprisingly, the calcineurin/NFAT signaling pathway also inversely regulates miR-133 expression. Calcineurin or miR-133 could regulate their own expression through a positive feedback mechanism, and cardiac hypertrophy is regulated by the reciprocal repression between miR-133 and calcineurin (Dong et al., 2010). In detail, either exogenous miR-133 supplement or endogenous miR-133 increase could give rise to the increase of transcriptional inhibition of calcineurin and suppression of NFAT, causing the increase of the miR-133 level. As a consequence, the increased miR-133 will further enchance miR-133 expression. By contrast, the downregulation of miR-133 could cause the increase of calcineurin transcription. Eventually, the activation of calcineurin induces the further activation of calcineurin/NFAT pathway (Figure 4; Dong et al., 2010).

#### RhoA/ROCK pathway

Notably, c-jun and c-fos constitute two subunits of activating protein-1 (AP-1), which is a transcriptional complex required for genes expression associated with cardiac hypertrophy; therefore, the development of cardiac hypertrophy is suppressed by the negative regulation of AP-1-binding activity (Wenzel et al., 2001). Similar to Cdc42, RhoA is also a member of the Rho subfamily of small GTP-binding proteins. The Rho-kinase (ROCK or Rho-associated coiled-coil protein kinase) family includes ROCK1 and ROCK2, serine/threonine kinases that phosphorylate a number of downstream substrates. RhoA stimulates the expression of c-jun and c-fos via ROCK and actin treadmilling, respectively (Na et al., 2012). The RhoA/ROCK signaling pathway participates in skeletal cell contraction and a variety of other cellular processes involving chondrogenesis and neuritogenesis by modulating the assembly of the actin cytoskeleton, which has also been confirmed to regulate cardiac hypertrophy (Yang et al., 2016; Cheng et al., 2017). In mice induced by TAC, miR-133 expression is significantly decreased in hypertrophic cardiomyocytes, and the restoration of its expression significantly represses hypertrophy through the RhoA/ROCK signaling pathway, indicating that RhoA is also a specific target of miR-133 in cardiomyocytes (Carè et al., 2007). However, the studies did not illustrate which pathway (ROCK1 or ROCK2) miR-133 affected to control hypertrophic growth because ROCK1 and ROCK2 exert relatively different physiological effects on cells. ROCK1 is required for the formation of stress fibers and the maturation of focal adhesion complexes. In contrast, ROCK2 inhibits the maturation of focal adhesions and stress fibers (Shi et al., 2013). In fibroblasts, ROCK1 knockdown leads to an absolute loss of polarity and the perturbation of peripheral actomyosin networks; however, the depletion of ROCK2 results in a clearly defined front and rear polarity (Lock et al., 2012; Newell-Litwa et al., 2015; Priya et al., 2017). Thus, further research is required to prove the hypothesis.

#### Inositol 1,4,5-triphosphate receptor

**Figure 5:**
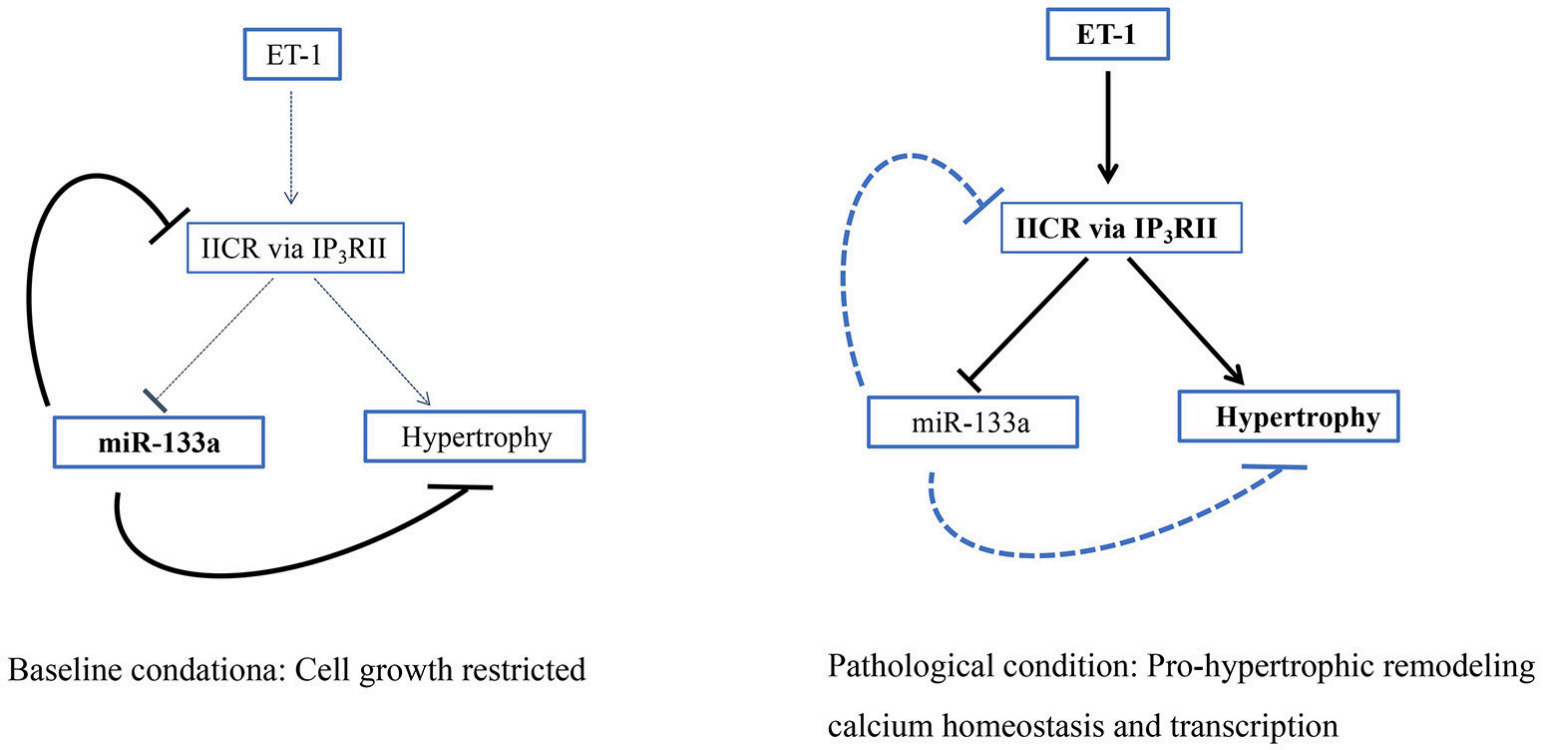
IICR participates in a pro-hypertrophic positive feedback loop. Schematic showing how the loss of miR-133a-mediated IP3RII inhibition generates a positive feedback loop to drive the hypertrophic response (Drawnel et al., 2012).

Inositol 1,4,5-triphosphate receptor (IP_3_R) II is a type of intracellular Ca^2+^ release channel that is mainly distributed in the nucleus and membrane of cardiomyocytes (Baker et al., 2017). IP_3_RII expression is obviously increased in hypertrophic cardiomyocytes induced by TNF-α and the hypertrophic, failing myocardium (Wang G. J. et al., 2017). IP_3_R and ryanodine receptors (RyR), but not L type Ca^2+^ channels, are the major calcium channels involved in the ectopic release of Ca^2+^ from intracellular Ca^2+^ stores in the sarcoplasmic reticulum. The ectopic Ca^2+^ released from these receptors induces the expression of pro-hypertrophic genes and may cause arrhythmias, playing important roles in the remodeling mechanisms (Sankar et al., 2014; Wang D. et al., 2017). MiR-133a constitutively restrains the expression of IP_3_RII during the hypertrophic response to neurohormonal stimuli or pressure overload, potentially promoting the physiological growth of cardiomyocytes until the balance between IP_3_RII and miR-133a is upset by pathological stimuli that could trigger inositol 1,4,5-triphosphate receptor (IP_3_)-induced calcium release (IICR). Intriguingly, when IICR is engaged in this fashion, sustained repression of miR-133a promoted by enhanced IICR generates a deleterious positive feedback loop, providing a powerful driving force for pathological cardiac remodeling (Figure 5; Drawnel et al., 2012). Briefly, miR-133a is promising target to protect against pro-hypertrophic transcriptional responses mediated by IICR because it inhibits IP_3_RII expression and IICR.

#### PI3K/AKT signaling pathway

The preceding section has elaborated the vital importance and mechanisms by which the PI3K signaling pathway participates in the development of cardiac fibrosis. In fact, in addition to the effects on cardiac fibrosis, the PI3K pathway plays more important and essential roles in regulating cardiomyocyte survival, cardiomyocyte size, angiogenesis, and inflammation during physiological and pathological cardiac hypertrophy (Braz et al., 2014).

Distinct signaling mechanisms associated with IGF-1-induced hypertrophy require Akt and its downstream molecules GSK-3β and mTOR. Overexpression of either IGF receptor or IGF-1 activates PI3K (p110α) will result in physiological cardiac hypertrophy and even MI, while a PI3K (p110γ) deficiency prevents HF induced by isoproterenol (Aoyagi and Matsui, 2011). In the mouse model, an IGF-1 deficiency protects against abdominal aortic constriction-induced cardiac hypertrophy by dampening Akt signaling and glucose transporter 4 (GLUT4) levels. However, overexpression of miR-133a and miR-1 also abrogates IGF-1-mediated cardiomyocyte hypertrophy and cardiac dysfunction (Hua et al., 2012). In addition, miR-133a directly targets AKT and alleviates cardiac hypertrophy by inhibiting the synthesis of translation-related proteins associated with hypertrophy by blocking PI3k/AKT/(mTOR, GSK3β) (Sang et al., 2015). However, because of the multiple downstream effectors in the PI3K signaling pathway and the diverse pathophysiological characteristics of hypertrophy, further studies are required to develop effective therapeutic approaches based on miR-133.

#### β-adrenergic receptors

The most effective mechanism to improve cardiac output in response to exercise or stress is to stimulate cardiomyocyte adrenergic receptors with noradrenaline released from the sympathetic nervous system (Charkoudian et al., 2010). The cAMP-dependent PKA signaling pathway primarily mediates the positive chronotropic, inotropic, and lusitropic effects of cAMP on cardiomyocytes, aiming to phosphorylate and modulate a number of core proteins involved in cardiac excitation-contraction coupling (Parks et al., 2017). In cardiac hypertrophy, chronically increased catecholamine levels induce cardiac β-AR signaling to desensitize the heart, accompanied by a reduction in cAMP-cyclic nucleotide phosphodiesterase and altered modulation of the β-AR/cAMP signaling pathway (Abi-Gerges et al., 2009). Similar to fibrosis, miR-133 also exerts a favorable effect on inhibiting β-AR in cardiomyocytes, the mechanisms of which may be related to the β-AR/ERK1/2 and cAMP signaling pathways (Wang D. et al., 2017). Additionally, the associations of miR-1/miR-133a, cAMP signaling pathway, hypertrophy and electrical remodeling were also evaluated in the mouse model of MI. Enhanced expression of the cAMP early repressor suppressed the expression of miR-1 and miR-133a, leading to hypertrophy and electrical remodeling, respectively. Meanwhile, by delivering miR-1 and miR-133a *in vivo*, inducible cAMP early repressor expression was blocked so that both hypertrophy and electrical remodeling were alleviated (Myers et al., 2015). Therefore, feedback mechanisms for cardiac-specific miR133a, miR-1, and cAMP signaling pathways exist in cardiac hypertrophy and electrical remodeling, providing proof-of-concept for the potential applications of miR-1 and miR-133a therapy for MI. However, in contrast to the aforementioned results, another study found that postnatal overexpression of miR-133a in cardiomyocytes does not affect reactive cardiac hypertrophy induced by isoproterenol or pressure overload, although it alleviates cardiac fibrosis (Matkovich et al., 2010). From our perspective, the possible explanations for the discrepancies in these results are mainly attributed to the use of different conceptual approaches in experimental models, the developmental stage at which miR-133a is expressed, and the time-point when samples are collected from transgenic models. For example, one study halted the normal downregulation of miR-133a in an acute hypertrophic stimulus model but did not completely knock out miR-133.

**Figure 6:**
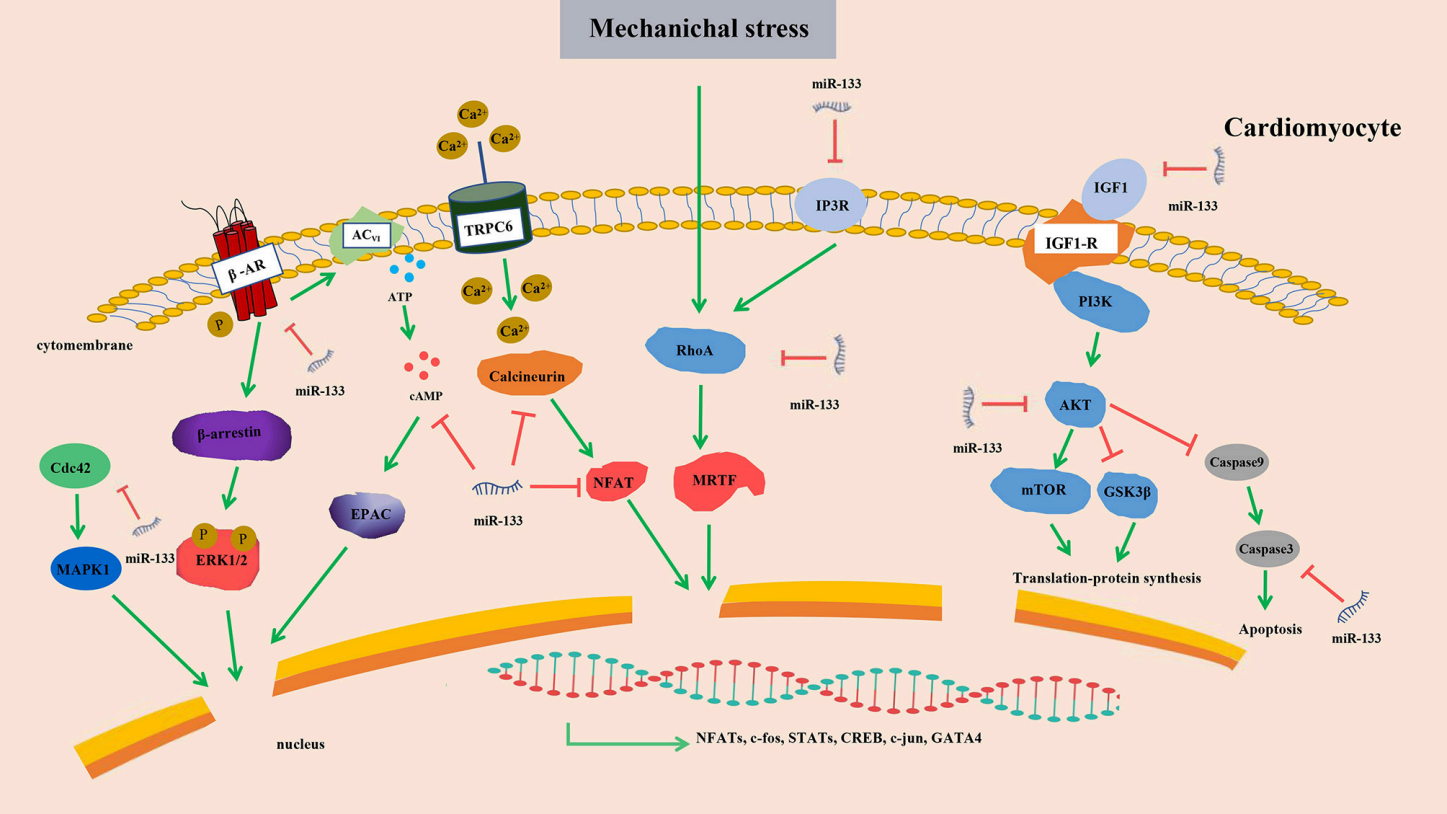
Targets of miR-133 in cardiomyocytes. Upregulated miR-133 inhibits the expression of β-AR, cAMP, Cdc42, AKT, Calcineurin, IP3R, RhoA, IGF1, and Caspase 3 and contribute to the activity of transcription factors, resulting in cardiac hypertrophy. β-AR, β-adrenergic receptor transduction; cAMP, 3′,5′ cyclic adenosine monophosphate; AKT, serine/threonine kinase; InoIP3R, sitol 1,4,5-triphosphate receptor; IGF-1, Insulin-like growth factor-1; MAPK, mitogen-activated protein kinase; ERK, extracellular signal-regulated kinases; EPAC, exchange protein directly activated by cAMP; NFAT, nuclear factor of activated T cell; mTOR, mammalian target of rapamycin; TRPC6, transient receptor potential 6; MRTF, myocardin-related transcription factor A; GSK3β, Glycogen synthase kinase-3β; Red arrows, suppression; green arrows, promotion.

The links between these hypertrophy-related miRNAs and corresponding targets or signaling pathways were briefly summarized (Figure 6). After considering all findings presented in this section, we postulate that miR-133 is very likely to suppress cardiac hypertrophy. The links between these hypertrophy-related miRNAs and corresponding targets or signaling pathways are highly complex. Hence, we may only be able to verify crucial regulatory nodules that can be manipulated to affect the outcomes by concentrating on network interactions rather than the signaling molecules themselves.

### A double-edged sword in cardiac electrical remodeling

Electrophysiological remodeling in HF and AF involves alterations in both the active membrane properties of heart cells and network attributes of the myocardium, which displays the hallmarks of prolonged action potential duration (APD) and slower conduction (John et al., 2008; Walmsley et al., 2013). The main pathogenesis resulting in prolonged APD and slower conduction include the downregulation of K^+^ currents and changes in depolarizing Na^+^ and Ca^+^ currents and transporters (Aslani et al., 2016). As the interactions between miRNAs and cardiac electrical remodeling are gradually unveiled, more and more miRNAs are being explored. For instance, miR-328 contributes to the adverse atrial electric remodeling in AF, the mechanism of which may be mediated by targeting L-type Ca^2+^ channel genes such as CACNA1C and CACNB1 (Lu et al., 2010). In cardiomyocytes from patients with persistent AF, the expression of predicted targets of miR-30d, including CACNA1C encoding Cav1.2 and KCNJ3 encoding Kir3.1, are also decreased, subsequently reducing the acetylcholine-sensitive inward-rectifier K^+^ current (Morishima et al., 2016). Currently, the relationship between miR-133 and cardiac electrical remodeling remains unclear, and controversy exists regarding whether miR-133 promotes or inhibits electrical remodeling.

Repolarization is modulated by a delicate balance between the inward and outward currents in cardiomyocyte membrane (Schroder et al., 2015). I_Kr_ and I_K1_, two key repolarizing currents, are mainly responsible for the repolarization of the membrane potential, which were mediated by the K^+^ channel subunits encoded by KCNJ2 and Ether-a-go-go related gene (ERG), respectively (Georgieva et al., 2008; Park et al., 2013). In the diabetic hearts, miR-133a regulates the expression of the ERG-encoded I_Kr_ channel, resulting in synchronous alterations at levels of the ERG mRNA and protein. The miR-133-mediated suppression of the ERG-encoded I_Kr_ channels leads to pathological QT prolongation. In this process, the level of miR-133 was also regulated by its upstream modulator SRF, which constituted a hyperglycaemia/SRF/miR-133/ERG/I_Kr_ axis to modulate the current in cardiomocytes (Xiao et al., 2011). Under some certain pathological conditions, such as arsenic trioxide (As_2_O_3_) exposure, I_Kr_ and I_K1_ may be blocked and contribute to a prolonged APD and QT interval (Chiang et al., 2002). A remarkable up-regulation of miR-1 and miR-133 was observed in the guinea pig model of As_2_O_3_-induced QT prolongation. The transfer of miR-133 into normal pig cardiomyocytes via a direct intramuscular injection prolonged the QT interval and increased the animals' mortality. Meanwhile, the expression of both the ERG protein and I_Kr_ was inhibited. Obviously, As_2_O_3_-induced cardiotoxicity in guinea pig hearts, including AF and sudden cardiac death, may be mediated by the upregulation of miR-133 and the inhibition of ERG and Kir2.1 K^+^ channel at the post-transcriptional level. The proposed molecular pathway is As_2_O_3_/SRF/miR-133/(KCNH2, ERG, and I_Kr_)/(APD and QT prolongation)/sudden cardiac death (Shan et al., 2013). In the clinic, plasma miR-133 levels in pediatric patients with ventricular tachycardia are obviously increased compared with normal children, which may also be attributed to damage to these repolarization current channels (Sun et al., 2015). However, further investigations are needed to validate this hypothesis.

Nevertheless, miR-133 has also been reported to exert a protective effect on cardiac electrical remodeling. Hyperpolarization-activated non-specific cation channels (HCNs) conduct a mixed K^+^/Na^+^ depolarizing current, which is activated by cAMP and hyperpolarization. Once the HCNs are activated in the myocardial diastolic period, membrane depolarization is triggered immediately (Myers et al., 2015; Wen and Li, 2017). In the right atrial appendage from patients with AF, the levels of HCN2 and HCN4 channels are significantly increased, accompanied by a decrease in miR-133 and miR-1 levels with aging. The mechanisms underlying this phenomenon may be that miR-133 and miR-1 exert inhibitory effects on HCN2 and HCN4 which could enhance the funny current and increase the incidence of atrial tachycardia and premature ventricular beats (Li et al., 2015). However, the protective mechanisms and targets of miR-133 were not extensively investigated in this study. What is more, by preventing the downregulation of miR-133a in a mouse model of TAC-induced cardiac remodeling, the downregulation of Kv4-encoded I_to, f_ was abolished despite the presence of abnormal repolarization, because miR-133 potentially enhanced the expression of Kv4-encoded I_to, f_ (Kcnip2), whose upregulation may decrease the content of collagen in fibroblasts and prevent abnormal I_to, f_ (Matkovich et al., 2010).

This section mainly generalizes the dual effects of miR-133 on cardiac electrical remodeling. We presume that miR-133 acts as a double-edged sword by activating or repressing the expression of different genes encoding membrane electrical channels. The number and activity of electrical channels directly determine the protective or destructive role in cardiac remodeling. Meanwhile, due to the complexity and specificity of the signal transduction network, the verification of any molecules as absolutely “good” or “bad” is imprudent and impossible. Hence, the precise mechanisms require further investigation.

### Promoting cardiac reprogram and maintaining cardiomyocytes

Cardiomyocytes with normal physiological functions are essential and critical to the heart, but in the failing heart, the number of normal cardiomyocytes is limited (Massengill et al., 2016). Thus, researchers have utilized numerous approaches to generate new cardiomyocytes, such as transplanting stem cells or inducing their differentiation (Zwi-Dantsis et al., 2013). MiRNAs are a group of intriguing candidates to regulate heart regeneration, given their abilities to simultaneously influence various mRNA targets (Eulalio et al., 2012). The upregulation of some cardiac-specific transcription factors and miR-133 in the heart can help to reprogram cardiac fibroblasts into cardiomyocyte-like cells (iCMs) with functional properties such as spontaneous calcium oscillations, L-type channel expression, and contractility (Muraoka et al., 2014). In mouse embryonic fibroblasts, the overexpression of miR-133 and Tbx5 generated seven-fold more beating iCMs and reduced the time to induce beating cells from 30 to 10 days compared with cells in which Tbx5 was up-regulated alone. In this reprogram process, miR-133 directly represses the expression of Snai1, a master regulator of the EMT. Intriguingly, this process by which the miR-133/Snai1 pathway silences fibroblast signatures is similar to the mesenchymal-to-epithelial transition (MET), a vital step in the reprogram of fibroblasts into induced pluripotent stem cells (iPSCs) by Yamanaka factors (Li R. et al., 2010; Samavarchi-Tehrani et al., 2010). Additionally, a combination of miR-133 and JAK inhibitor I treatment exhibits a significant improvement in efficiency by enhancing either the expression of cardiac ion channels or α-MHC (Jayawardena et al., 2012). Mesenchymal stem cells (MSCs) are a class of pluripotent, adult stem cells that are easily expanded in culture and can differentiate into several mesenchymal cell lineages, including vascular endothelial cells and iCMs (Soleymaninejadian et al., 2015). In the ischaemic microenvironment of the heart, the transfection of miR-133a in MSCs significantly improves MSC survival and engraftment by inhibiting the expression of the Caspase-9 and Apaf-1 mRNAs, subsequently resulting in decreased cell apoptosis and cardiac fibrosis and improved cardiac function (Dakhlallah et al., 2015). Furthermore, in cardiac progenitor cells (CPCs) obtained from adult mouse hearts, miR-133a overexpression protected CPCs from apoptosis and increased their capacity to inhibit hypertrophy by targeting the proapoptotic genes Bmf and Bim, but the proliferation and differentiation potential were not affected (Izarra et al., 2014).

In H9c2 cells, miR-133 and miR-1 exert opposite regulatory effects on cardiomyocyte apoptosis induced by oxidative stress. When miR-1 and miR-133 were co-transfected into cardiomyocytes, oxidative stress-induced apoptosis was not affected. However, in the presence of oxidative stress, the levels of miR-133 and miR-1 were both markedly increased compared to cells that were not exposed to oxidative stress. Notably, a substantially greater increase in miR-1 expression was observed than in miR-133 levels, indicating miR-133 is an anti-apoptotic factor while miR-1 is a pro-apoptotic factor. The underlying mechanism is probably that miR-133 targets Caspase 9 and miR-1 targets heat shock protein (HSP) 60 and HSP70 to inhibit apoptosis (Xu et al., 2007; Friedrich et al., 2015). Similarly, in a rat model of ischaemia-reperfusion injury, ischaemic post-conditioning protects against cardiomyocyte apoptosis by upregulating miR-133 to target the caspase cascade-mediated apoptotic pathway (He et al., 2011).

Notably, miR-133a-deficient hearts display increased and aberrant cardiomyocyte proliferation throughout the atria and ventricles, which potentially explains the development of a lethal ventricular septal defect in miR-133 knockout mice (Liu et al., 2008). Liu et al. investigated the cardiac transcriptome of miR-133a knockout hearts to identify the mechanism by which miR-133a regulates cardiomyocyte proliferation and confirmed the up-regulation of several transcription factors involved in cell cycle control, namely, Cyclin D1, Cyclin D2, and Cyclin B1. Among these factors, Cyclin D2 contained a miR-133a seed sequence in its 3′-UTR, indicating that Cyclin D2 may serve as a direct target of miR-133 (Liu et al., 2008). Consistent with findings from mammals, in a zebrafish model subjected to heart resection surgery, miR-133 inhibited cardiomyocyte proliferation by modulating the activity of monopolar spindle 1 (Mps1) and Cx43. However, in the absence of cardiac injury, cardiomyocyte proliferation was not influenced significantly, regardless of the expression levels of miR-133 (Yin et al., 2012). Furthermore, miR-133 also plays a key inhibitory role in the vascular smooth muscle cell phenotypic switch by suppressing the expression of the transcription factor Sp-1 *in vitro* and *in vivo*. In this regulatory pathway, the level of miR-133 was determined by extracellular signal-regulated kinase 1/2 activation (Torella et al., 2011).

Taken together, an HF therapy must maintain the normal proportion and functions of cardiomyocytes in the heart. Gratifyingly, cardiac fibroblasts that have been reprogrammed into iCMs and stem cells show a promising future in cardiomyocyte regeneration. Hence, further studies are needed to explore the precise mechanisms underlying the functions of miR-133 in reprogram.

In addition to its direct cardioprotective effects mediated by the four mechanisms discussed throughout this review, miR-133 is also linked to energy balance and might indirectly prevent cardiac remodeling. For instance, miR-133 could decide the cell commitment between brown adipocytes and myocytes by targeting Prdm16, indicating that miR-133 may be a promising therapeutic target for diabetic cardiomyopathy associated with obesity (Trajkovski et al., 2012).

## Diagnostic and therapeutic potential of miR-133 in cardiac remodeling: future perspectives

Over the last several years, several studies conducted using animal models have uncovered revealed the potential of miRNAs for the diagnosis and treatment of a vast array of diseases, ranging from cardiovascular diseases to neurological diseases and cancer (Lu et al., 2008). For instance, in patients with HF undergoing the implantation of a left ventricular assist device, reduced serum miR-133a levels are significantly associated with increased Beclin 1, LC3B, and ATG3 expression, thus resulting in the exacerbation of diabetes-induced cardiac autophagy and hypertrophy (Nandi et al., 2015). In surgical patients with coronary artery disease, decreased expression of miR-133 also significantly correlates with an increased severity of HF (Danowski et al., 2013). Lower miR-133 levels have been detected in peripheral blood mononuclear cells (10.16 ± 4.81 vs. 37.03 ± 8.18, *p* < 0.05) from patients with left ventricle diastolic dysfunction compared with the healthy controls (Marketou et al., 2013). Actually, in addition to stressing the excellent potential of miRNAs as biomarkers of cardiovascular diseases, several studies have also highlighted new challenges and possibilities for the development of innovative therapeutic strategies using either transgenic or gene deletion approaches. One strategy is to utilize exogenous drugs to restore or suppress the expression of miR-133 by regulating the activity of its upstream and downstream molecules. Currently, certain drugs with preclinical relevance have been already proven to regulate miR-133 expression both *in vitro* and *in vivo*. For example, carvedilol protects cardiomyocytes from H_2_O_2_-induced apoptosis by up-regulating miR-133, although the study did not provide any insights into the mechanism by which carvedilol regulated miR-133 levels in response to oxidative stress (Xu et al., 2014). Additionally, a choline supplement inhibited TAC-induced cardiac hypertrophy by restoring the abnormally downregulated expression of miR-133a and the calcineurin protein (Zhao et al., 2013). Moreover, HOP, histone deacetylases and the JAK inhibitor mentioned above are also promising drugs for resetting the epigenome and reprogram cardiac remodeling. The intake of a high-salt diet might result in cardiac fibrosis in individuals with salt-sensitive hypertension by suppressing miR-133a expression; therefore, the intake of a low-salt diet, in essence, is also an exogenous regulator of miR-133 expression (Guo et al., 2014). Additionally, other endogenous and exogenous molecules, such as adiponectin (Abi-Gerges et al., 2009) and hydrogen sulfide (H_2_S) (Mishra et al., 2012), have also been shown to upregulate miR-133 in the heart to protect against cardiac hypertrophy and diabetic cardiomyopathy. Another strategy, which may be the most advanced and feasible therapeutic strategy (at least in experimental animal models), is to employ miR-133 mimic or its antagonist as molecular drugs to directly target corresponding genes and proteins involved in cardiac remodeling. Although substantial progress has been achieved in miRNA-based therapeutics, researchers are still confronted with several issues with the precise delivery of drugs to the heart and the efficient targeting of specific cell populations. Further investigations are required to prevent the degradation of synthetic miR-133 and to determine its pharmacokinetics and pharmacodynamics in the human body. Therefore, we conjecture that the clinical applications of vector-based gene therapy for cardiac remodeling will be likely to be introduced in the very near future.

## Conclusions

In this review, we described the roles and mechanisms of miR-133 in cardiac remodeling, which might be beneficial references for clinical applications and future investigations. Importantly, miR-133 inhibits the progression of cardiac hypertrophy and cardiac fibrosis, promotes the reprogram of cardiac fibroblasts into iCMs, improves MSC survival and represses CPC apoptosis, all of which indicate that miR-133 serves as a weapon against cardiac remodeling. Intriguingly, miR-133 may act as a double-edged sword in cardiac electrical remodeling. It alleviates arrhythmia by regulating the expression of some ion channels; meanwhile, it also aggravates electrical activity disorders, causing AF and sudden death. Thus, miR-133 functions through diverse target genes and sophisticated signaling pathways in cardiac remodeling, including RhoA, Cdc42, HERG, and PI3K/Akt signaling pathways. Although numerous investigations are exploring the relationship between miR-133 and various cardiac disorders, we hypothesize that additional potential target genes and molecular pathways of miR-133 will be identified in subsequent investigations.

More empirical data pertaining to the improvements in cardiac function and the safety and efficacy of various HF models are required before miR-133 is translated from the bench to the bedside. We sincerely hope that this review provides some insights into future clinical applications and further studies.

## Author contributions

NL was responsible for conceiving and designing the review and writing the manuscript. HZ was in charge of conceiving and designing some portions of the review and writing the manuscript. HZ reviewed and edited the manuscript. QT was the principal investigator of this study and serves as the corresponding author.

### Conflict of interest statement

The authors declare that the research was conducted in the absence of any commercial or financial relationships that could be construed as a potential conflict of interest.

## Abbreviations

HF: Heart failure
miRNAs: MicroRNAs
Ang II: Angiotensin II
MI: Myocardial infarction
ECM: Extracellular matrix
α-SMA: α-smooth muscle actin
EMT: Epithelial-mesenchymal transition
MET: Mesenchymal-to-epithelial transition
AF: Atrial fibrillation
β-AR: β-adrenergic receptor transduction
CTGF: Connective tissue growth factor
SRF: Serum response factor
Col1A1: Collagen 1A1
TAC: Transverse aortic constricted
IP_3_RII, Inositol 1, 4: 5-triphosphate receptor II
IICR: IP_3_-induced calcium release
NFAT: Nuclear factor of activated T cells
IGF-1: Insulin-like growth factor-1
ERG: Ether-a-go-go related gene
As_2_O_3_: Arsenic trioxide
MET: Mesenchymal-to-epithelial transition
iPSCs: Induced pluripotent stem cells
3′-UTR: 3′-untranslated region
TGF-β: Transforming growth factor-β
MSCs: Mesenchymal stem cells
iCMs: Cardiomyocyte-like cells
CPCs: Cardiac progenitor cells
HSP: Heat shock protein
UTR: Untranslated regions
Mef2: Myocyte enhancer factor-2
MyoD: Myogenic differentiation-1
APD: Action potential duration
Mps1: Monopolar spindle 1
cAMP, 3′: 5′ cyclic adenosine monophosphate
MAPK: mitogen-activated protein kinase
ERK: Extracellular signal-regulated kinases
NFAT: nuclear factor of activated T cell
TGF-β: transforming growth factor β
HCNs: Hyperpolarization-activated non-specific cation channels.
